# Detection of the inferred interaction network in hepatocellular carcinoma from EHCO (Encyclopedia of Hepatocellular Carcinoma genes Online)

**DOI:** 10.1186/1471-2105-8-66

**Published:** 2007-02-27

**Authors:** Chun-Nan Hsu, Jin-Mei Lai, Chia-Hung Liu, Huei-Hun Tseng, Chih-Yun Lin, Kuan-Ting Lin, Hsu-Hua Yeh, Ting-Yi Sung, Wen-Lian Hsu, Li-Jen Su, Sheng-An Lee, Chang-Han Chen, Gen-Cher Lee, DT Lee, Yow-Ling Shiue, Chang-Wei Yeh, Chao-Hui Chang, Cheng-Yan Kao, Chi-Ying F Huang

**Affiliations:** 1 Institute of Information Science, Academia Sinica, Taipei 115, Taiwan, R. O. C; 2 Department of Life Science, Fu-Jen Catholic University, Taipei Hsien 242, Taiwan, R. O. C; 3 Division of Molecular and Genomic Medicine, National Health Research Institutes, Miaoli County 350, Taiwan, R. O. C; 4 Institute of Cancer Research, National Health Research Institutes, Taipei 114, Taiwan, R. O. C; 5 Department of Computer Science and Information Engineering, National Taiwan University, Taipei 106, Taiwan, R. O. C; 6 Institute of Biomedical Science, National Sun Yat-Sen University, Kaohsiung 804, Taiwan, R. O. C; 7 National Center for High-performance Computing, Hsinchu 300, Taiwan, R. O. C; 8 Institute of Bio-Pharmaceutical Sciences, National Yang-Ming University, Taipei 112, Taiwan, R. O. C; 9 Institute of Biotechnology in Medicine, National Yang-Ming University, Taipei 112, Taiwan, R. O. C

## Abstract

**Background:**

The significant advances in microarray and proteomics analyses have resulted in an exponential increase in potential new targets and have promised to shed light on the identification of disease markers and cellular pathways. We aim to collect and decipher the HCC-related genes at the systems level.

**Results:**

Here, we build an integrative platform, the Encyclopedia of Hepatocellular Carcinoma genes Online, dubbed EHCO , to systematically collect, organize and compare the pileup of unsorted HCC-related studies by using natural language processing and softbots. Among the eight gene set collections, ranging across PubMed, SAGE, microarray, and proteomics data, there are 2,906 genes in total; however, more than 77% genes are only included once, suggesting that tremendous efforts need to be exerted to characterize the relationship between HCC and these genes. Of these HCC inventories, protein binding represents the largest proportion (~25%) from Gene Ontology analysis. In fact, many differentially expressed gene sets in EHCO could form interaction networks (e.g. HBV-associated HCC network) by using available human protein-protein interaction datasets. To further highlight the potential new targets in the inferred network from EHCO, we combine comparative genomics and interactomics approaches to analyze 120 evolutionary conserved and overexpressed genes in HCC. 47 out of 120 queries can form a highly interactive network with 18 queries serving as hubs.

**Conclusion:**

This architectural map may represent the first step toward the attempt to decipher the hepatocarcinogenesis at the systems level. Targeting hubs and/or disruption of the network formation might reveal novel strategy for HCC treatment.

## Background

Hepatocellular carcinoma (HCC) is the most common liver malignancy and is one of the leading causes of death worldwide. Its incidence has been especially prevalent among Asian populations. Due to HCC being the top cause of cancer death worldwide, research on its cause, diagnosis, and treatment continues into the post-genomic era. However, the carcinogenesis of HCC still remains poorly understood.

In the post-genomic era, advances in tools and technologies have provided an excellent opportunity to better understand the complex interaction of hepatocarcinogenesis. For example, genome-wide microarray technologies, which are widely used to monitor global gene expression in cancer [1-14], have identified numerous differentially expressed genes and enable cancer research to succeed where traditional methods have faltered. These high-throughput analyses have revolutionized the way that HCC is diagnosed and classified. It is generally believed that microarrays are well able to shed light on the identification of disease markers for diagnosis and potential targets for treatment. However, there are several bottlenecks associated with moving from microarray profiling to target identifications. The challenges include: (1) infrastructural challenges, such as the creation of data models and databases for storing data, the integration of data with external databases and the extraction of information from natural language text; (2) information overload, where there are many HCC-related microarray studies [1-14], the gene annotations are scattered across different databases and the content is difficult to update or improve. Moreover, an exponential growth in both the number and the size of specialized biological databases has also made the task of performing cross-site browsing or iterative querying very tedious and challenging. There have been several attempts, such as GeneWebEx [39] and GENA [40] to integrate cross-site information, but these often lack adequate annotation related to HCC. All this calls for the establishment of an infrastructure that can collect such scattered annotations, present them in a user-friendly way, and allow viewers to participate actively in its making.

To embrace the paradigm shift to the next generation of the web and to provide web-based services, we have designed an information harvesting infrastructure, Encyclopedia of Hepatocellular Carcinoma genes Online, dubbed EHCO, which employs softbots (or Web wrapper agents [15]) to collect scattered gene annotations either by mining data sources directly or by querying publicly accessible databases. To overcome the obstacles that EHCO reports merely descriptive results from various studies, we notice that protein bindings from Gene Ontology analysis represent the largest proportion (~25%) in the molecular function category. By using available protein-protein interaction datasets, a highly interactive biological network and novel hubs can be revealed among these seemingly random HCC inventories.

## Results and Discussion

### The architecture of EHCO

**Figure 1:**
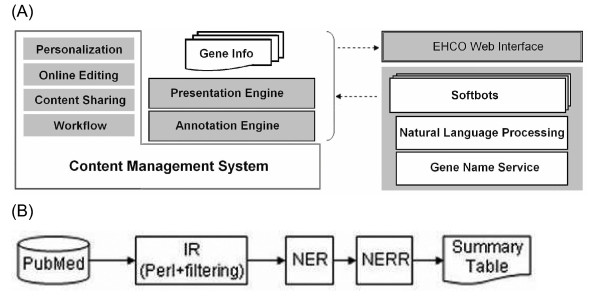
**The architecture of EHCO**. (A) EHCO uses a Content Management System, PLONE, to maintain different types of information. PLONE supports workflow design, content sharing, front-end editing, and member registration. Softbots, which interact with a software environment by using and interpreting the environment feedback, are used as annotation collectors to retrieve scattered genomic information across the Internet. EHCO also implements Natural Language Processing, a subfield of artificial intelligence and linguistics, and Gene Name Service, a comprehensive cross reference service of all widely used gene ID nomenclatures, to support the annotation engine, which is supported by mySQL database and python-written scripts. The Presentation Engine uses Wiki pages to allow dynamic information display as well as user commenting. (B) We performed biological information retrieval (IR) to obtain PubMed abstracts that may contain gene-HCC relationships, followed by two information extraction tasks: (1) Named Entity Recognition (NER): to recognize biomedical named entities (NEs) and (2) Named Entity Relation Recognition (NERR), as shown in the flowchart.

EHCO adapts the PLONE platform, an open source content management system (CMS) with a workflow engine, pre-configured security and a set of content types, to create an infrastructure to support flexible storage and presentation (Figure 1A). The advantage of using PLONE is that PLONE supports Wiki so that EHCO can be extended beyond the context of HCC research. One example is Liver Fibrosis [41], a sister site of EHCO, which provides liver necroinflammatory and fibrosis-related gene knowledge [38]. Both sites shared the same server, same PLONE, the same mySQL database, and most of the python codes. The uniqueness of EHCO lies in its ability to allow registered users to contribute their own work to EHCO to create an integrated biological information portal for efficient information sharing and extensive aggregation of research-related topics. To achieve the uniqueness, EHCO uses ZWiki as its content management and presentation platform. ZWiki is one of many open-source Wiki platforms freely available online. With ZWiki, users can collaboratively create their new Web pages to enrich the contents of EHCO.

### The differentially expressed gene set collections in EHCO

**Figure 2:**
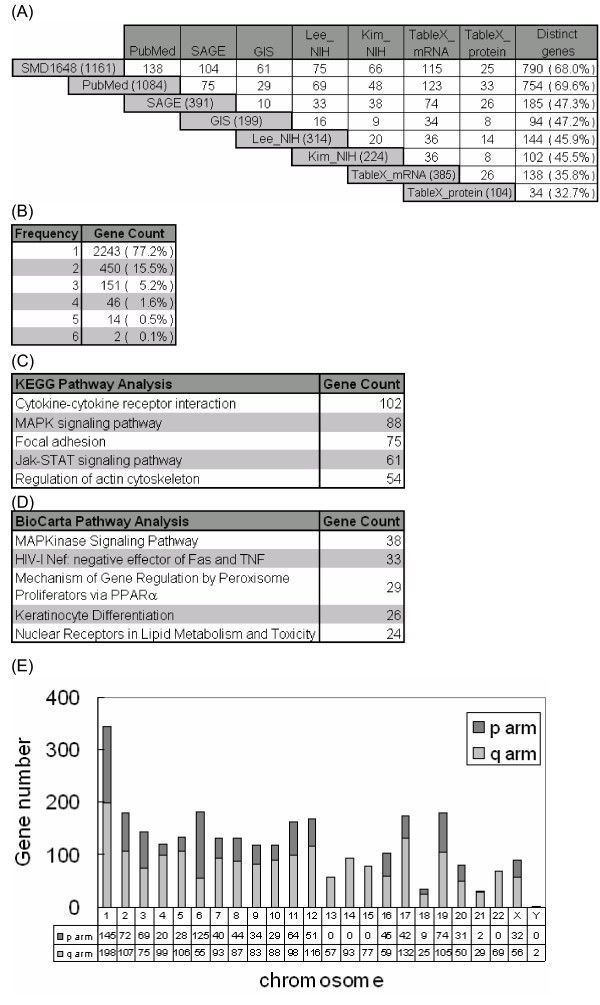
**Statistics analysis of EHCO collections**. (A) Gene intersections among the 8 different gene sets collected in EHCO. The numbers in the parenthesis are the gene numbers in each dataset. The intersection between each dataset is shown in the box, e.g. SMD1648 has 138 genes in common with PubMed. (B) Distribution of HCC-related genes in EHCO. (C, D) Top 5 pathways, as analyzed by KEGG and BioCarta pathway collectors, associated with HCC-related genes in EHCO. (E) Chromosomal distribution of HCC-related genes in EHCO.

A fundamental part of EHCO is the collection of eight gene sets related to HCC either from PubMed or diverse high-throughput studies (Figure 2A). Since different labs obtained all gene sets independently at different time, they used a wide variety of gene IDs and appeared in many cases with different gene names, resulting in difficulty in gene ID comparisons. Therefore, we established Gene Name Service [49], a comprehensive gene name cross-reference database, to unify all of the aliases automatically. Since the amount of biomedical literatures available on the web is rapidly increasing, manual information extraction from search results is usually unable to identify articles of interest immediately. Therefore, in the PubMed section, we had extracted 1,084 genes (with HUGO-approved gene names) from approximately 4,500 abstracts in the PubMed category (Figure 1B) (detail in Methods section). For those genes that either do not have HUGO-approved gene names or fail to be included from PubMed search, they were placed under TableX when reading the articles. Among the HCC-related microarray studies, EHCO was further reorganized into five gene sets. Differentially expressed HCC-related gene sets were collected from four major studies, including Chen et al., [1] (referred to as SMD1648), Neo et al., [2] (referred to as GIS), Lee et al., [3] (referred to as Lee_NIH), and Kim et al., [4] (referred to as Kim_NIH), and ten additional reports [5-14], which were manually keyed in and are referred to as TableX_mRNA. The differentially expressed genes collected from these sets ranged from 199 (GIS) to 1,161 (SMD1648). Similarly, eight proteomics reports [16-23], which represented relatively small collections of 104 proteins, were also manually keyed in and are referred to as TableX_protein. In addition, the SAGE dataset [48] was collected from CGAP library by using a 2-fold difference in tumor *vs*. non-tumor sample as criteria, resulting in a gene set of 391 genes.

### Intersection genes

**Figure 3:**
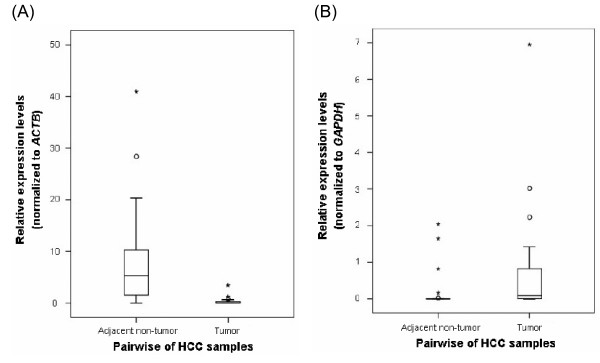
**Validation the EHCO dataset by quantitative RT-PCR**. (A) NNMT is down-regulated and (B) PEG10 is overexpressed in HCC. The mRNA expression levels of NNMT and PEG10 were determined by quantitative RT-PCR in 21-pairwised HCC patients. Results were normalized against the mRNA expression level of ACTB (A) and GAPDH (B) in each sample.

Among the eight gene set collections, there are 2906 non-redundant genes in total. Figure 2A shows the intersection between each gene set. The biggest intersection, which contained 138 genes, appeared between SMD1648 and PubMed. Interestingly, 68.0% SMD1648 (790 out of 1,161) and 69.6% PubMed (754 out of 1,084) collections (referred to as distinct genes in Figure 2A) had not been reported in other gene sets. Similarly, a comparison was made that revealed 77% genes were only counted once among each gene set (Figure 2B), suggesting that tremendous efforts will be needed to characterize the relationship between HCC and these genes generated from diverse measurements. A cross-dataset comparison of SAGE and five microarray datasets (mRNA-based measurements) revealed the top 23 intersection genes, which appeared at least 4 times in EHCO, among each gene set. 19 out of the 23 genes shared consistent gene expression patterns (tumor *vs*. normal; up- or down-regulated) (shown as Up & Down) (Table 1). In contrast, 4 genes had discrepancies in HCC expression patterns (bold in Table 1). This might be the result of changes in gene expression associated with different pathophysiological states, the type of sample collected, etc. Fortunately, three out of four had PubMed records, including FABP1 (overexpression in HCC [24]), IGFBP3 (down-regulation in HCC [25]), and SGK (overexpression in HCC [10]). Since we did not have an unbiased method to distinguish the accuracy of NNMT, Q-RT-PCR was applied to evaluate the expression patterns by using 21 pairwised HCC patient specimens. The down-regulation profiles were observed in 19 out of 21 paired samples analyzed (Figure 3A). In addition to NNMT, we chose PEG10 [26,27], which also had contradictory expression patterns in our collections, and performed Q-RT-PCR to validate the gene expression signatures. Figure 3B showed that overexpression of PEG10 was observed in 17 out of 21 HCC patient samples. Altogether, the majority of EHCO collections (~77%) appeared only once and there were some discrepancies among gene sets, indicative of a need for an immediate further validation of these different measurements by using different HCC samples. Therefore, we especially welcome other investigators to contribute their validation data to our EHCO data warehouse.

**Table 1:** Top 23 genes that appear most frequently in 6 mRNA-related gene sets in EHCO.

Count	Symbol	SMD1648	SAGE	GIS	LEE	KIM	TableX_mRNA
5	SCP2	Down	Down		Down	Down	Down

4	ADH1B	Down	Down	Down			Down
	ALB	Down	Down			Down	Down
	ARG1	Down	Down			Down	Down
	CAT	Down	Down		Down	Down	
	CP	Down	Down			Down	Down
	CPB2	Down	Down			Down	Down
	CRHBP	Down		Down		Down	Down
	CYP2C9	Down	Down			Down	Down
	CYP2E1		Down	Down	Down		Down
	FGB	Down	Down			Down	Down
	GHR	Down		Down		Down	Down
	HPD	Down	Down		Down		Down
	HSD17B6	Down	Down			Down	Down
	MT1B	Down		Down	Down	Down	
	PCK1	Down	Down		Down		Down
	PHYH	Down			Down	Down	Down
	TF	Down	Down			Down	Down
	TTR	Down	Down		Down		Down
	FABP1	Down	Down			Down	**Up**
	IGFBP3	Down	**Up**	Down			Down
	NNMT	Down	Down			Down	**Up & Down**
	SGK	Down	Down			Down	**Up**

### Pathway and chromosome assignments

Pathway information is important for an understanding of the functionality of genes and proteins. EHCO integrated two well-known pathway databases, KEGG [28] and BioCarta [51]. Figure 2C and 2D show the top five KEGG and BioCarta pathways that have the most HCC-related genes associated with them in these two public accessible pathway collections. Out of EHCO's current collections, 2385 genes were identified as part of the associated pathways from KEGG. The most associated pathway is cytokine-cytokine receptor interaction, which has 102 genes associated with it. The most associated pathway in BioCarta is the MAPK signaling pathway, which has 38 genes associated with it. Interestingly, these two public accessible pathway analyzers did not have a similar dataset for their top 5 collections except for the MAPK signaling pathway. This is probably due to the fact that the primary areas of KEGG collections are metabolic pathways whereas the coverage areas of BioCarta are signaling pathways. Moreover, analysis of chromosome distribution indicates that the largest proportion belongs to chromosome 1 (343 genes), whereas Y chromosome contains the least genes, namely only 2 EHCO genes (Figure 2E).

### Harvesting gene annotations through softbots and weblinks

The disease specific gene/protein expression pattern can provide important clues about gene function. Currently, ~1000 genes-related to HCC are in our PubMed collections (or referred to as small scale studies). To date, EHCO has extended the HCC-related gene collections to 2,906 genes. The study of these genes can be accelerated by functional annotations.

To organize diverse HCC-related datasets, the annotation handler stepped in to annotate the EHCO collected gene sets. We use Softbots [29] to harvest gene annotations from various web resources. A softbot is an intelligent software robot that acts, on behalf of the user, to achieve certain goals. Given the resources, which can be online websites, databases, or documents, a softbot extracts the information that it has been targeted to. Since softbot can be fragile against changes to the online resources, we used a Java program, called Agent Toolbox [15] to create and maintain these softbots. Agent Toolbox 'learns' the information extraction rules from users' labels on the web page with a machine-learning algorithm [36,37]. As a result, we can efficiently repair and maintain our softbots. In this study, individual softbots were used to mine different targets. These programs will periodically update the latest information from NCBI databases and download them into EHCO. Finally, we created a presentation engine integrating all the information into a single user-friendly page view as "Gene Info" (Figure 1A). As a result, each gene annotation webpage displays the most up-to-date information from various databases, e.g. EBI [52], and SMART [30].

To rapidly interrogate the expression patterns of our collection, two gene sets (SMD1648 and PubMed) were used as templates to annotate our EHCO collections. Firstly, SMD-HCC dataset reports not only those differentially expressed gene in SMD1648, but also records the expression patterns of other genes (~16,000 genes), which are not statistically significant in the original data analysis. Inclusion of this on-line quantitative evaluation of gene expression provides a rapid visualization of global trends in gene expression. Secondly, we applied a natural language processing technique to extract information from the abstracts, followed by a summary table illustrating the gene and disease relationship. Toward this goal, we have developed a tool, as described in the Method section, to annotate genes, diseases, and other HCC-related information (i.e. HBV, HCV) from PubMed abstracts to provide a brief summary of each EHCO genes.

### Detection of inferred biological network of HCC

**Figure 4:**
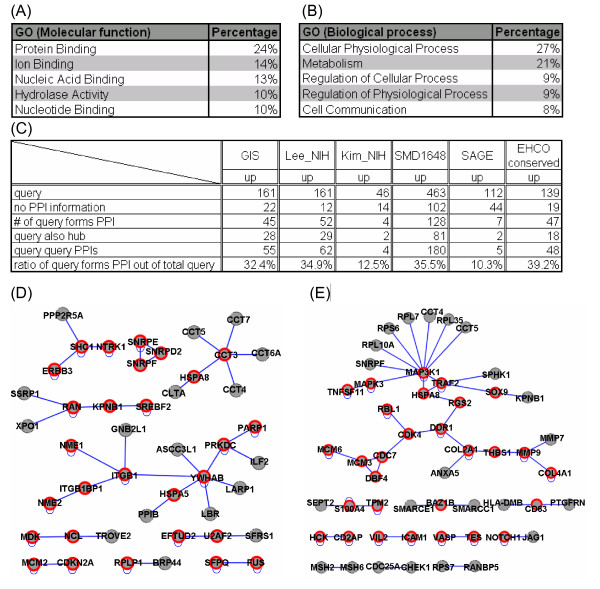
**Detection of inferred biological network of HCC**. (A, B) Gene Ontology (GO) analysis reveals top 5 categories in molecular function and biological process. Protein binding is the largest proportion in molecular function (A), whereas cellular physiological process ranks number one in the biological process (B). (C, D, E) Detection of protein-protein interaction (PPI) network from various gene sets. Only up-regulated genes (referred to as queries) in each gene set are subjected to search for their interaction partners. Many queries can form interaction network among query themselves, despite the fact that many queries do not have PPI data. (D) Of 139 GIS queries, 45 of them can form 55 PPI to constitute the interaction networks and 28 (red circle) of them serve as hubs (referred to as interacting with more than one query, including homo-dimer). (E) Of 149 Lee_NIH queries with available PPI data, 52 of them (34.9%) can interact with each other and form 62 PPIs.

The availability of HCC-related gene catalogs now enables elucidation of molecular mechanisms governing hepatocarcinogenesis at the systems level. However, these gene catalogs give no direct clues, at least not immediately evident from the gene symbols, to the underlying carcinogenesis processes. Gene Ontology (GO) analysis [42], which allows the identification of functional related clusters, indicates that protein binding from molecular function (Figure 4A) and cellular physiological process from biological process (Figure 4B) represent the largest proportion (approximately 25%), respectively, in either category. This analysis raises the possibility that these HCC-related genes might not act alone randomly, but have a propensity to associate physically or functionally to perturb the fundamental biological processes of the liver.

To uncover the potential interaction networks or synergistic effects of these seemingly unrelated HCC-related genes, we employed each up-regulated gene set as queries and searched for their interaction partners by accessing our previously established protein-protein interaction (PPI) data [31,32]. Figure 4C summarizes the analysis results. We used GIS dataset as the first example because all tissue samples in the study were HBV-associated HCC patients. GIS consists of 161 overexpressed genes, but 22 of them have no PPI information. Of the remaining 139 queries, 45 of them (32.4%) could interact with each other (Figure 4C and 4D) and form 55 PPIs. Moreover, 28 queries serve as "hubs" (Figure 4D label with red circle), which are referred to as queries interact with more than one query in a given PPI network (see later). Next, we used Lee_NIH as the second example because this study was using mouse model to recapitulate the HCC and may provide novel insight toward the hepatocarcinogenesis. Similarly, of 149 queries with available PPI data, 52 of them (34.9%) can interact with each other to constitute 43 PPIs and 29 hubs can be revealed (Figure 4E label with red circle). Both queries in the inferred networks belong to primarily cellular physiological process and metabolism from biological process in GO. Together, transformation of these seemingly random genes into their corresponding PPI reveals the intrinsic dys-regulated biological network in HCC and highlights the potential new hubs for discovery of the potential HCC markers or therapeutic targets.

### Evaluation of "hubs" in the conserved HCC network at the systems level

**Figure 5:**
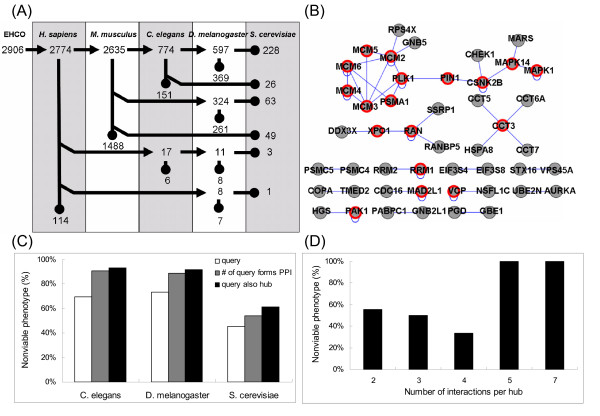
**An architectural map of the conserved HCC network at the systems level**. (A) 228 HCC-related genes in EHCO collections are evolution conserved cross various species. EHCO collects 2906 HCC-related *H. sapiens *genes, of which 2,635 genes have *M. musculus *homologs (point by arrow) and 114 genes cannot find any homologs (point by node). Of 2635 *M. musculus *genes, there are 774 genes with *C. elegans *homologs and 324 genes have *D. melanogaster *homologs, but do not have *C. elegans *homologs (branch and point by arrow). In short, 228 genes are conserved from *H. sapiens *to *S. cerevisiae*. (B) Conserved HCC network. Among 139 overexpressed and evolutionary conserved HCC-related genes, 120 of them have at least one PPI record. 47 of them can interact with each other and constitute a network and 18 of them are query-also-hubs (red circle). (C) Evaluation of the essentialness of hubs. To evaluate the essentialness of hubs, 139 queries, 47 queries constituting of interaction network, and 18 query-also-hubs were subjected to search for the nonviable phenotype in their corresponding homologs in various model organisms, including *C. elegans*, *D. melanogaster*, and *S. cerevisiae*. (D) Evaluation of the feature of hub degree. The degree, which is referred to as number of interactions associated with a protein, is considered to be one of the features in determining the essentialness of hubs. 18 query-also-hubs, which have various degrees ranging from 2–7, were subjected to search for the nonviable phenotype in their corresponding homologs in *S. cerevisiae*. The relative ratio is shown.

It has been illustrated using the yeast PPI network that this network is scale-free, in which some proteins have many more interactions than others [33]. A survey of the yeast protein interaction network has revealed that proteins with more interactions are more important than those with fewer interacting proteins [34], suggesting that hub degree is an indicator for essentialness in a network. However, it is difficult to address the essentialness of these newly identified hubs (or potential new targets for hepatocarcinogenesis) from the inferred biological network as described in Figure 4D and 4E. Therefore, we employed the comparative genomics approach for the availability of knockout phenotype from various model organisms. To reduce the complexity, we focused on those genes only conserved in evolution, which may represent, at least in part, the dys-regulation of essential cellular physiological roles in hepatocarcinogenesis as concluded in GO analysis (Figure 4B). Based on the HomoloGene orthologues database (build50.1), 228 genes, consisting of 139 up- and 78 down-regulated genes, out of 2906 EHCO collections are conserved range from *H. sapiens*, *M. musculus*, *C. elegans*, *D. melanogaster*, to *S. cerevisiae *(Figure 5A). Of 139 queries, 120 queries with PPI data were individually subjected to search for their interaction partners. 47 out of 120 queries (~40%) were associated with each other and there were 18 hubs (Figure 5B, labeled in red) in this network. By using the available phenotypic information from WormBase [44], FlyBase [45], and SGD [46], the percentage of nonviable phenotypes, e.g. embryonic or larval lethality, or sterility, in these 18 hubs was higher than those 120 queries (Figure 5C). Moreover, of these 18 hubs with more interaction proteins, the ratio of nonviable phenotype in yeast seemed to be higher than those with fewer interaction proteins (Figure 5D). This analysis seems to be able to shed light on which research can be based to study potential new players in HCC research in the post-genomic era through the systems biology approach.

## Conclusion

The collected genes or associated pathways in EHCO may represent only small part of the whole genome or our understanding of hepatocarcinogenesis. This network construction supporting the view that well coordinated interaction networks may be required for these aberrant HCC-related genes to modulate hepatocarcinogenesis. Targeting multiple hubs and/or disruption of the network formation could offer an excellent opportunity to reveal potential strategy for HCC treatment.

## Methods

### Text-mining to acquire HCC-related gene sets

The fundamental part of EHCO is the collections of eight gene sets related to HCC. The text-mining method to acquire HCC-related literatures from PubMed used "hepatocellular carcinoma" as a keyword. This study used the latest approved human genome nomenclature from HUGO Gene Nomenclature Committee [47]. A simple text-matching program was written to look for the presence of HUGO-approved gene names, symbols, and aliases, in the title and abstract part of HCC-related literatures, resulting in acquiring approximately 24,300 abstracts. Because one publication may contain several genes, we curated the results by removing redundancy and obtained 10,425 abstracts. These potential HCC-related abstracts and gene lists were further verified to remove unrelated abstracts by natural language processing, followed by manual read through by biology major graduate students, research associates and postdoctoral fellows. Finally, in the PubMed category, we had extracted 1,084 genes from 4,492 abstracts.

### Information retrieval and extraction by natural language processing

To elucidate the relationship between the EHCO collected genes and HCC, we applied a natural language processing (NLP) technique to extract information from the literature. We performed biological information retrieval (BioIR) to obtain PubMed abstracts that may contain gene-HCC relationships, followed by information extraction to reveal gene-HCC relationships. BioIR started with searching for keyword combinations, i.e. (gene symbol or aliases) and (hepatocellular carcinoma). 10,425 PubMed abstracts were scanned to search for HCC candidate genes. We filtered out abstracts irrelevant to gene-HCC relationships by eliminating those abstracts that HCC and genes did not co-occur in the same sentence since most NLP techniques only handled information in the same line. 5,942 abstracts were found to contain gene-HCC relationships. Biology majored associates were manually read through the abstracts, resulting in the validation of 4,492 abstracts. The accuracy (or precision in NLP terminology) is about 75%. Error analysis on the output and retrieved abstracts indicates that most of errors were caused by lack of sufficient syntactic patterns. The retrieved relevant PubMed abstracts were then subjected to automated information extraction.

To extract information related to a certain topic, the extraction tool needs to be capable of accomplishing two tasks: (1) Named Entity Recognition (NER): to recognize biomedical named entities (NEs) (e.g. afp, alb, mRNA and HCC); and (2) Named Entity Relation Recognition (NERR): to recognize relationships of interest between NEs (e.g. HCC and mRNA level). NER in biomedical domain is a challenging problem in natural language processing for the following reasons. Most biomedical named entities have no nomenclature. They may include long compound words (e.g. hepatocellular carcinoma), or unknown words that include hyphen, digit, Greek letter, or Roman numeral (e.g. 4'-mycarosyl isovaleryl-CoA transferase). Abbreviations and acronyms may also appear as unknown words and frequently cause ambiguity in their meanings. Moreover, named entities may involve variations in spelling (e.g. N-acetylcysteine, N-acetyl-cysteine, NacetylCysteine) or in expressions (e.g. EGF receptor and EGFR). To handle this NER problem, we constructed a NE dictionary that contains the following NEs and NE classes along with their aliases: gene, protein, mRNA, serum, hepatitis B virus (HBV), hepatitis C virus (HCV), methylation, liver regeneration, HCC, cirrhosis, fibrosis, and necrosis. We used dictionary-based method, i.e. a dictionary and a fast matching algorithm, to identify NEs of our interest occurring in the PudMed abstracts. Next, we proceeded to investigate the relationship between NEs by using a natural language parser to assign part-of-speech annotation, and co-occurrences of terms, and template-based methods. The overall automated procedure to extract gene-HCC relationships for our gene-HCC knowledge base system is shown in Figure 1B.

### Patients and tumor samples

21 pairs of tumor and adjacent non-tumor liver tissues were collected from 1996 to 2000 at the Division of General Surgery of Kaohsiung Veterans General Hospital (VGH). No patients had previously received any treatment, e.g. chemotherapy, for HCC. The study protocol had the approval of the ethics committee at Kaohsiung-VGH. All patients gave informed consents. Study samples, including tumor and adjacent non-tumor liver tissues, were obtained during diagnostic biopsy, and non-tumor liver tissues were derived from neighboring site outside of the tumor. Both tumor and adjacent non-tumor liver tissues for subsequent studies were confirmed by pathologists and were used for quantitative RT-PCR (or Q-RT-PCR). The detailed protocols for RNA isolation and SYBR Green I based quantitative RT-PCR were as described previously [35].

## Availability and requirements

Project name: Functional genomics analysis elucidates the signaling networks of hepatocellular carcinoma.

Project home page: 

Operating system(s): Platform independent.

Any restrictions to use by non-academics: no licence needed.

## Authors' contributions

CNH, JML, and CYFH designed the project, analyzed the results and wrote the paper. CHL, HHT, KTL, GCL, DTL and CWY designed the architecture of EHCO and implemented softbots to harvest gene annotation. TYS and WLH were responsible for natural language processing part of the project. LJS and CHC did the Q-RT-PCR experiment. SAL and CYK did the network construction. HHY, YLS, and CHC were responsible for database collections and manually curation of the collected information. All authors read and approved the final manuscript.
